# Mitochondria as biosynthetic factories for cancer proliferation

**DOI:** 10.1186/s40170-015-0128-2

**Published:** 2015-01-25

**Authors:** Christopher S Ahn, Christian M Metallo

**Affiliations:** Department of Bioengineering, University of California, San Diego, La Jolla, CA 92093 USA; Moores Cancer Center, University of California, San Diego, La Jolla, CA 92093 USA

**Keywords:** Cancer, Mitochondria, Biosynthesis, Amino acids, Nucleotides, Anaplerosis, Lipogenesis

## Abstract

Unchecked growth and proliferation is a hallmark of cancer, and numerous oncogenic mutations reprogram cellular metabolism to fuel these processes. As a central metabolic organelle, mitochondria execute critical biochemical functions for the synthesis of fundamental cellular components, including fatty acids, amino acids, and nucleotides. Despite the extensive interest in the glycolytic phenotype of many cancer cells, tumors contain fully functional mitochondria that support proliferation and survival. Furthermore, tumor cells commonly increase flux through one or more mitochondrial pathways, and pharmacological inhibition of mitochondrial metabolism is emerging as a potential therapeutic strategy in some cancers. Here, we review the biosynthetic roles of mitochondrial metabolism in tumors and highlight specific cancers where these processes are activated.

## Review

**Figure 1:**
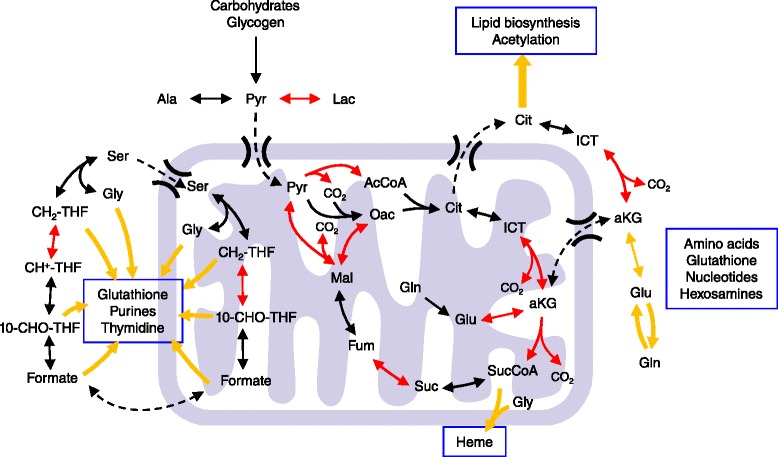
**Biosynthetic nodes within mitochondria.** Metabolic pathways within mitochondria that contribute to biosynthesis in cancer and other proliferating cells. TCA metabolism and FOCM enable cells to convert carbohydrates and amino acids to lipids, non-essential amino acids, nucleotides (including purines used for cofactor synthesis), glutathione, heme, and other cellular components. Critical biosynthetic routes are indicated by yellow arrows. Enzymatic reactions that are dependent on redox-sensitive cofactors are depicted in red.

Recent characterizations of metabolic enzymes as tumor suppressors and oncogene-driven metabolic reprogramming have reinvigorated interest in cancer metabolism. Although therapies targeting metabolic processes have long been a staple in cancer treatment (e.g. inhibition of folate metabolism via methotrexate), the focused therapeutic potential surrounding these findings have generated a renewed appreciation for Otto Warburg’s work almost a century ago. Warburg observed that tumor cells ferment much of the glucose taken up during growth to lactate, thus using glycolysis as a major means of adenosine triphosphate (ATP) regeneration [1]. However, the observation of decreased respiration in cancer cells and idea that “*the respiration of all cancer cells is damaged*” belies the critical role of mitochondria in biosynthesis and cell survival [1]. On the contrary, functional mitochondria are present in all proliferative cells within our body (including all tumors), as they are responsible for converting the diverse nutrients available to cells into the fundamental building blocks required for cell growth. These organelles execute numerous functions in cancer cells to promote tumor growth and survival in response to stress. Here, we outline the critical biosynthetic functions served by mitochondria within tumors (Figure 1). Although many of these functions are similarly important in normal, proliferating cells, we have attempted to highlight potential points where mitochondrial metabolism may be therapeutically targeted to slow cancer growth. This review is organized by specific metabolic pathways or processes (i.e., glucose metabolism and lipogenesis, amino acid metabolism, and nucleotide biosynthesis). Tumors or cancer cell types where enzymes in each pathway have been specifically observed to by dysregulated are described within the text and summarized in Table 1.

**Table 1:** **Overview of mitochondrial biosynthetic enzymes important in cancer**

	**Cancer type (according to primary site)**											
	**Blood, bone, or lymph**	**Brain**	**Breast**	**Colon**	**Kidney**	**Liver**	**Lung**	**Stomach**	**Ovarian**	**Pancreas**	**Prostate**	**Skin**
*TCA cycle, anaplerosis, and AcCoA metabolism*												
Pyruvate carboxylase		**•**	**•**	**•**	**•**	**•**	**•**			**•**		**•**
Pyruvate dehydrogenase complex	**•**	**•**	**•**	**•**	**•**		**•**			**•**		**•**
Isocitrate dehydrogenase (activity or mutation)	**•**	**•**	**•**	**•**	**•**	**•**	**•**			**•**		**•**
Succinate dehydrogenase (mutation)					**•**							
Fumarate hydratase (mutation)					**•**							
Glutaminase and/or glutamate dehydrogenase	**•**	**•**	**•**	**•**	**•**	**•**	**•**			**•**		**•**
Glutamine synthetase		**•**	**•**			**•**						
*Amino acid metabolism*												
Pyrroline-5-carboxylate reductase	**•**	**•**	**•**	**•**	**•**		**•**		**•**	**•**	**•**	**•**
Proline oxidase	**•**	**•**	**•**	**•**	**•**	**•**	**•**	**•**	**•**	**•**	**•**	**•**
Aspartate transaminase			**•**			**•**				**•**		
Alanine transaminase				**•**		**•**	**•**	**•**		**•**		**•**
*Nucleotide biosynthesis*												
Dihydroorotate dehydrogenase	**•**	**•**	**•**	**•**	**•**		**•**				**•**	**•**
Methylenetetrahydrofolate dehydrogenase	**•**	**•**	**•**	**•**		**•**	**•**	**•**	**•**	**•**	**•**	**•**

Cancers in which three or more mitochondrial enzymes have been studied and found to be differentially regulated (or mutated, as indicated) in cancers vs. control groups are included. Dysregulation of each enzyme was demonstrated in clinical tumors samples, animal models, or cell lines at the levels of genes, mRNA, protein, metabolites, and/or flux.

### Glucose anaplerosis

Glucose is the most widely available nutrient in our body; not surprisingly, most tumor cells consume this carbohydrate (or analogs) at high rates. This phenotype allows for detection and imaging of some cancers and metastatic lesions using the glucose analog 2-deoxy-2-[^18^ F]fluoro-D-glucose (FDG), which accumulates in tumors (and some other tissues) and can be noninvasively observed when using positron emission tomography integrated with computed tomography (FDG-PET/CT) [2]. While FDG-PET/CET tracks cells with high glucose uptake and phosphorylation only, the use of isotope tracers, mass spectrometry (MS), and nuclear magnetic resonance (NMR) have enabled researchers to more closely examine the fate of glucose within cancer cells [3-5]. Indeed, flux through glycolysis and lactate secretion remains a hallmark of many tumor cells, presumably to facilitate biosynthesis of ribose, purines (via serine and glycine), and lipid headgroups (via glycerol-3-phosphate and serine). However, increasing evidence now indicates that cancer cells transport a significant portion of glucose-derived pyruvate into mitochondria where it serves as an anaplerotic substrate to replenish tricarboxylic acid (TCA) cycle intermediates used for biosynthesis. For example, conditions of limited glutamine availability or glutaminase (GS) suppression drive cancer cells to increasingly rely on glucose carbon flux through pyruvate carboxylase (PC) to maintain oxaloacetate (OAC) production and downstream TCA cycle activity [6]. Furthermore, NMR analysis of mice bearing three distinct human orthotopic tumors and infused with [3,4-^13^C_2_]glucose indicated these glioblastoma lines used glucose as a mitochondrial anaplerotic substrate [7]. Although glutamine is one of the most abundant amino acids present in plasma, typical *in vitro* culture media used for cell line expansion contain relatively high concentrations (2–4 mM) of glutamine. Thus, as tumor cells are increasingly analyzed prior to “adaptation/selection” *in vitro*, we are beginning to better appreciate the importance of glucose-derived pyruvate as an anaplerotic substrate in tumors.

### Glucose oxidation and lipogenesis

Beyond flux through PC or analogous reactions, the more predominant fate of mitochondrial pyruvate is oxidation by the pyruvate dehydrogenase (PDH) complex to form acetyl-coenzyme A (AcCoA). AcCoA is subsequently converted to citrate via condensation with OAC by citrate synthase. In turn, citrate is either converted to isocitrate in the TCA cycle or transported out of mitochondria and metabolized by ATP citrate lyase to yield cytosolic AcCoA, which is the substrate for *de novo* lipogenesis and acetylation. Mitochondrial activity within this pyruvate-citrate shuttle is therefore critical for the biosynthesis of fatty acids and cholesterol as well as protein acetylation. With some notable exceptions (e.g. hypoxia, discussed below), most cancer cells derive the majority of their lipogenic AcCoA from glucose-derived pyruvate through PDH [8-10]. Numerous oncogenic pathways stimulate glucose-derived carbon atom flux through the citrate shuttle to promote lipogenesis and TCA metabolism. Specific mutations in Kirsten rat sarcoma viral oncogene homolog (*KRAS*) stimulate flux of glucose through PDH to generate fatty acids [11,12]. Alternatively, active Akt promotes glucose-mediated fatty acid synthesis downstream of PDH [8,13]. This Akt-dependent lipogenesis occurs by activation of mammalian target of rapamycin complex 1 (mTORC1) and sterol regulatory element-binding protein 1 (SREBP1), which are key regulators of cellular growth and lipid homeostasis, respectively [14]. Interestingly, SREBPs have also been shown to coordinate lipid and protein biosynthesis as well as protect cancer cells from saturated fatty acid-induced lipotoxicity [15,16]. On the other hand, inactivation of mTORC1 reduces mitochondrial fluxes that supply the citrate and AcCoA which fuel these pathways [17,18]. Finally, overexpression of the *HER2* oncogene or epidermal growth factor (EGF) stimulation both activate MEK/ERK signaling to suppress the inhibitory PDH kinase 4 (PDK4) and maintain glucose oxidation in mammary epithelial cells [19].

These above concepts and results contrast the established role of PDH kinase 1 (PDK1) in supporting tumor growth downstream of hypoxia-inducible factor (HIF) signaling by suppressing PDH activity [20-22]. Indeed, inhibition of PDK1 activity using dichloroacetate (DCA) forces glucose oxidation under hypoxic conditions [9] and inhibits the growth of xenograft tumors [23]. Limited mitochondrial glucose metabolism due to hypoxic or pseudohypoxic stabilization of HIFs is a hallmark of some renal carcinomas [9,24,25], and normalization of HIF levels (thus increasing glucose oxidation) in such cells abrogates tumor formation in xenografts [26]. Suppression of PDK1 to activate PDH flux also contributes to BRAF(V600E)-induced oncogene senescence [27], further suggesting that limiting glucose oxidation is important for tumor growth. Furthermore, some tumors downregulate expression of the mitochondrial pyruvate carrier (MPC), and acute inhibition of the MPC in cancer cells significantly decreases glucose oxidation but has no effect on growth or respiration [28-30]. Tumor cells are clearly able to compensate for this lack of glucose-mediated biosynthesis under these conditions through extramitochondrial pathways, scavenging acetate [31,32], unsaturated lipids [8,33], or proteins [34] when required. Therefore, the relative importance of glucose-driven biosynthesis through mitochondrial pathways may be tumor specific. Alternatively, there may be a particular level of glucose flux into mitochondria that supports biosynthesis while limiting oxidative TCA metabolism and potentially deleterious byproducts (e.g. reactive oxygen species; ROS). Further mechanistic studies are required to characterize the mechanisms through which cancer cells balance mitochondrial energetic (catabolic) and biosynthetic (anabolic) metabolism.

### Amino acid metabolism

**Figure 2:**
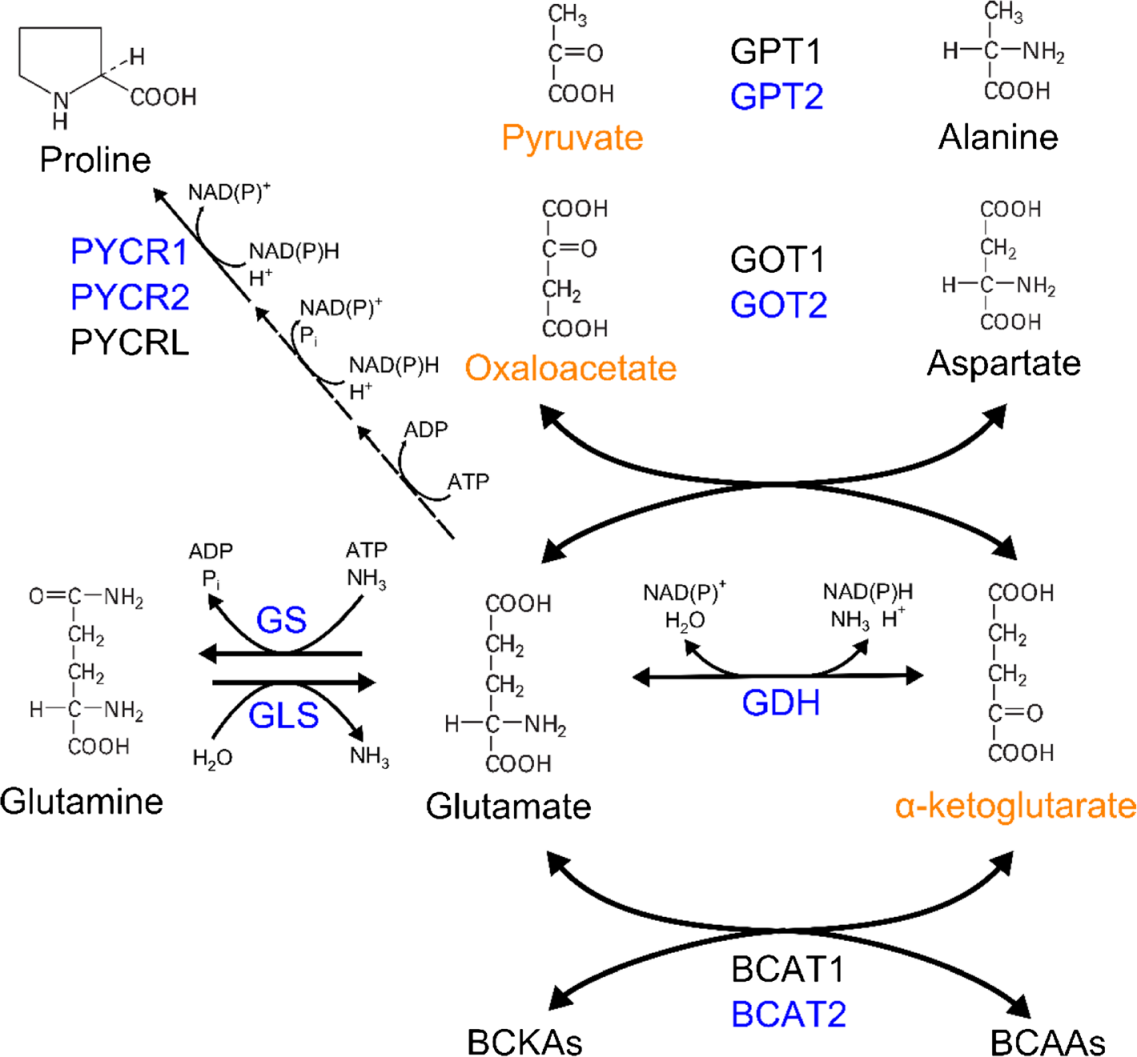
**Coordination of carbon and nitrogen metabolism across amino acids.** Glutamate and aKG are key substrates in numerous transamination reactions and can also serve as precursors for glutamine, proline, and the TCA cycle. Mitochondrial enzymes catalyzing these reactions are highlighted in blue, and TCA cycle intermediates are highlighted in orange (pyruvate enters the TCA cycle as acetyl-CoA or oxaloacetate).

In addition to carbohydrates, amino acids are critical substrates fueling mitochondrial metabolism and the biosynthesis of proteins, lipids, and other molecules. Of particular interest in cancer are key mitochondrial enzymes in the metabolism of glutamine, glutamate, proline, aspartate, and alanine (Figure 2). Glutamine is one of the most critical nutrients required for cell proliferation, as the amido nitrogen of this amino acid is the obligate substrate for hexosamine and nucleotide biosynthesis in the cytosol. Furthermore, the carbon backbone of glutamine is an important anaplerotic substrate fueling TCA cycle metabolism (Figure 1). Upon conversion to glutamate via glutaminase (GLS) activity, N-acetyl-glucosamine production, or nucleotide biosynthesis, glutamine carbons enter the TCA cycle as alpha-ketoglutarate (aKG) downstream of glutamate dehydrogenase (GDH) or transaminase activity [35,36]. The GLS (rather than GLS2) isoform is commonly expressed in tumors and is regulated downstream of the *MYC* oncogene [37]. Pharmacological inhibition of GLS is being investigated as a potential means of therapy for a number of different tumor types [38-40]. Indeed, GLS facilitates oxidative glutaminolytic flux in tumor cells derived from gliomas, lymphomas, breast cancers, prostate cancers, pancreatic cancers, and melanomas [38,40-44]. Recent flux studies in tumor cells bearing isocitrate dehydrogenase 1 (IDH1) mutations indicate that these cells may be particularly dependent upon glutamine to fuel oxidative mitochondrial metabolism and thus may be responsive to inhibition of GLS or respiration [39,45]. GLS-derived glutamate is also important for glutathione synthesis, which is abundant at mM levels in cells and plays an important role in redox homeostasis and tumor cell survival in response to oxidative stress [46].

#### Glutaminolysis and reductive carboxylation

Glutamine carbon can also fuel AcCoA generation for lipid biosynthesis when metabolized by malic enzymes (MEs) through glutaminolysis or alternatively via reductive carboxylation. The former pathway, by which glutamine-derived malate is converted to pyruvate and subsequently lactate or AcCoA, is active in some tumor cells that express high levels of cytosolic *ME1* or the other mitochondrial isozymes *ME2* and *ME3* [44,47]. Leukemic cells under hypoxia have been observed to employ this pathway for biosynthesis and ATP regeneration [38]. Glutaminolysis is also specifically activated in proliferating cells upon inhibition of MPC function, which may contribute to the sensitivity of cancer cells to inhibitors of glutamine metabolism [29,30].

In contrast to the oxidative glutaminolytic pathway, reductive carboxylation involves the “reverse” or reductive activity of NADP^+^-dependent IDHs to generate isocitrate and citrate from aKG, a pathway which becomes important in cells proliferating in hypoxic microenvironments or those with dysfunctional mitochondria [9,25,42,48]. HIFs stabilized by low oxygen levels or loss of the von Hippel Lindau tumor suppressor reduce PDH activity [20,21], leading cells to use alternate substrates for AcCoA generation such as glutamine or aKG [24]. In this manner, mass action and/or altered mitochondrial redox states induce proliferating cells to reductively metabolize aKG via NADP^+^-dependent IDHs and subsequently generate isocitrate and ultimately AcCoA [49]. Evidence also suggests that aKG-dehydrogenase (*OGDH*) and nicotinamide nucleotide transhydrogenase (*NNT*) expression are both required for activation of this pathway [48,50]. Indeed, hypoxic cells maintain and even upregulate oxidative glutamine metabolism in mitochondria despite the observed increase in reductive carboxylation activity [38,45,51]. Thus, some mitochondrial functions are required to allow conversion of glutamine to AcCoA through this pathway. On the other hand, cells with heterozygous mutations in IDH1 are specifically compromised in their ability to use reductive carboxylation for fatty acid synthesis [45], suggesting that the cytosolic isozyme catalyzes reductive carboxylation. While the specific contributions and functions of mitochondrial IDH2 and cytosolic IDH1 in this pathway must be definitively characterized (both *in vitro* and *in vivo*), increased exchange of aKG and isocitrate/citrate occurs in the context of perturbed redox states when fatty acid biosynthesis is maintained, a common occurrence in the tumor microenvironment. Ultimately, this pathway may effectively allow cancer cells to maintain biosynthesis, transfer reducing equivalents between compartments, or both to support growth and survival in hypoxic microenvironments.

#### Glutamine synthesis

Many amino acids are not extremely abundant in plasma or the tumor microenvironment and therefore must be synthesized *de novo*. Mitochondrial metabolism plays a definitive role in the production of many non-essential amino acids and their further utilization in biosynthetic pathways. Although glutamine is relatively abundant in plasma, *de novo* glutamine synthesis in the liver and surrounding tissues is likely critical for tumor cell growth. Glutamine biosynthesis requires a supply of aKG from mitochondrial metabolism to generate glutamate (a critical precursor for most non-essential amino acids) and subsequently glutamine via glutamine synthetase (GS). *De novo* glutamine biosynthesis in tumors has been detected *in vivo* using infusions of [^13^C]glucose into mice bearing human glioblastoma orthotopic tumors [7]. Furthermore, some breast epithelial cells can mediate glutamine independence via expression of *GS* [52]. Finally, glutamine as well as other amino acids may be scavenged via protein catabolism when it is not available in sufficient quantities [34].

#### Proline metabolism

Mitochondrial proline metabolism and synthesis are critically important for tumor cells, at least in part due to the unique, modifiable chemical properties it provides to proteins. Proline is synthesized from glutamine or urea-cycle-derived ornithine via the intermediate pyrroline-5-carboxylate (P5C). P5C is then converted to proline via the NAD(P)H-dependent enzyme pyrroline-5-carboxylate reductase (PYCR), which exists in three isoforms: PYCR1, PYCR2, and PYCRL (Figure 2). Mitochondrial PYCR1 and PYCR2 are upregulated in multiple types of cancer, including prostate, lymphoma, and others [41,53,54]. Overexpression of *c-Myc* in P493 human Burkitt lymphoma and PC3 human prostate cancer induced an upregulation of *PYCR1* expression as well as the P5C biosynthetic enzyme delta-1-pyrroline-5-carboxylate synthase (*P5CS*), resulting in higher levels of intracellular proline [41]. In line with this observation, expression of both *PYCR1* and *PYCR2* was increased in a panel of melanoma cell lines but was undetectable in normal melanocytes [53]. Furthermore, a recent large-scale comparative analysis of published mRNA microarray datasets found that *PYCR1* was one of the most commonly overexpressed metabolic enzyme genes in comparison to normal tissue among the 19 represented cancer types [54]. Although the functional advantages provided to cancer cells by modulating proline metabolism are not completely clear, the importance of proline in extracellular matrix proteins (e.g. collagen) could play a role in tumorigenesis. Alternatively, interconversions of proline and P5C in the cytosol and mitochondria have been proposed as a means of transferring reducing equivalents between these compartments [55], though more detailed functional analyses are required to elucidate how proline metabolism contributes to cancer progression.

Downregulation of proline catabolism is complementary to its biosynthesis and commonly observed in a number of tumor types. The first step of this process is catalyzed in the mitochondria by proline oxidase (POX), and the expression of this enzyme is markedly reduced in many cancers compared to normal tissue from the same patient [56]. *POX* expression is induced by the tumor suppressor p53, and ectopic expression of *POX* in DLD-1 colon cancer cells induces cell cycle arrest and reduces tumor burden in xenograft models [56]. Furthermore, *POX* expression is inhibited by MYC via miR-23b* in lymphoma, renal, and prostate cancers [41,57]. The widespread repression of POX in cancer indicates that this enzyme may act as a tumor suppressor; however, the specific mechanisms through which POX deficiency promotes tumorigenesis are not yet clear.

#### Aspartate and asparagine metabolism

**Figure 3:**
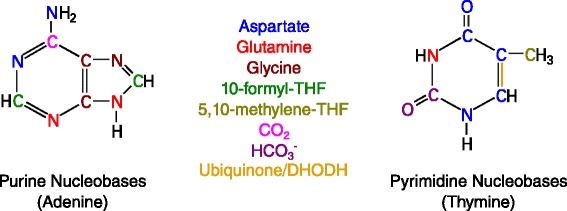
**Biosynthetic sources for purine and pyrimidine synthesis.** Sources and fates of nitrogen, carbon, and oxygen atoms are colored as indicated. Italicized metabolites can be sourced from the mitochondria or cytosol. The double bond formed by the action of DHODH/ubiquinone is also indicated.

Aspartate can be generated from the TCA intermediate oxaloacetate by glutamate-mediated transaminase activity (Figure 2); thus, the biosynthesis of aspartate and downstream metabolites is intimately tied to mitochondrial activity. Aspartate transaminases (GOT1, cytosolic; GOT2, mitochondrial), which bidirectionally convert aspartate and aKG to OAC and glutamate, are important for the growth of human pancreatic adenocarcinoma (PDAC) [43]. Oncogenic *KRAS*, the most common mutation in PDAC, redirects glutamine metabolism toward aspartate production in a number of settings [11,43,58]. This metabolic reprogramming is thought to facilitate regeneration of NADPH for reductive biosynthesis and redox homeostasis as well as NAD^+^ for maintaining glycolysis [43]. Ablation of oncogenic *KRAS* in a mouse model of pancreatic cancer markedly reduced tumor size and also revealed a subpopulation of surviving tumor cells which did not express *KRAS*. These surviving cells relied heavily on oxidative phosphorylation and were sensitive to oligomycin treatment, providing evidence that inhibition of mitochondrial function may effectively target cells that survive after suppression of oncogenic *KRAS* signaling [59]. Additionally, aspartate and glutamine are the precursors for asparagine, which is synthesized in the cytosol by asparagine synthetase (ASNS). *ASNS* expression is required for the survival of cultured glioma and neuroblastoma cell lines, and supplementation of exogenous asparagine can prevent apoptosis induced by glutamine withdrawal, in part, by modulating ER stress [60]. Expression of *ASNS* is also correlated with drug resistance in childhood acute lymphoblastic leukemia (cALL) and some forms of acute myeloblastic leukemia (AML), which are typically deficient in their ability to synthesize asparagine *de novo* [61,62]. Finally, aspartate is a key initiator of pyrimidine synthesis and donates nitrogen for purine synthesis via adenylosuccinate synthetase (Figure 3), further highlighting the role of mitochondrial aspartate metabolism in tumor cell biosynthesis.

#### Alanine and BCAA metabolism

Alanine production via alanine transaminases (GPT1, cytosolic; GPT2, mitochondrial), which transfer an amino group between glutamate and pyruvate to yield alanine and aKG, not only provide proteinogenic alanine but also aKG for TCA cycle activity (Figure 2). Maintenance of glutamine anaplerosis and catabolism in cancer cells via increased GPT2 activity is essential for oncogenic *KRAS*-induced anchorage independent growth, as demonstrated by knockdown of *GPT2* expression in HCT116 colon cancer cells [35]. GPT activity may also facilitate disposal of excess nitrogen (such as that derived from glutamine) via alanine secretion [63]. Indeed, secretion of alanine is higher in melanoma cell lines compared to normal melanocytes and is quite significant in human colon carcinoma tumors [35,64].

Finally, the branched chain amino acids (BCAAs) valine, leucine, and isoleucine are also highly metabolized by transaminases in both the cytosol (via BCAT1) and mitochondria (via BCAT2) (Figure 2) [65]. While cytosolic BCAT1 metabolism has been implicated in gliomas with wild-type IDH1 [66], how BCAA catabolism contributes to cancer progression remains unclear. Ultimately, by coordinating cellular bioenergetics and biosynthesis through the TCA cycle, amino acid metabolism plays a critical role in tumor growth and survival.

### Nucleotide biosynthesis

In addition to amino acid and lipid biosynthesis, nucleotide production is highly dependent upon mitochondrial metabolism and associated intermediates. While the ribose moiety of nucleotides is exclusively generated in the cytosol, many components that contribute to both pyrimidine and purine bases are derived directly or indirectly from mitochondria (Figure 3). Pyrimidine ring synthesis requires glutamine and aspartate, which can be supplied by mitochondrial pathways as noted above. Pyrimidine synthesis also requires the activity of dihydroorotate dehydrogenase (DHODH), a mitochondrial enzyme that converts dihydroorotate to orotate coupled with the reduction of ubiquinone to ubiquinol. Importantly, oxidation of ubiquinol in the electron transport chain (ETC) is necessary to maintain an adequate supply of ubiquinone for DHODH activity. In fact, uridine must be supplemented to culture media to allow proliferation of ρ^0^ cells (i.e., cells lacking functional mitochondrial DNA) and other cell lines with genetic modifications that compromise respiration [45,67]. Thus, DHODH links cellular respiration and pyrimidine synthesis. Elevated *DHODH* expression and increased activity have been observed in multiple types of cancers (Table 1) [68-71]. Inhibition of DHODH in human melanoma decreases growth both *in vitro* and in murine xenografts [70]. Doxorubicin, a common chemotherapeutic, induces a decrease in *DHODH* expression and acts synergistically with tumor necrosis factor-related apoptosis-inducing ligand (TRAIL) to selectively kill tumor cells [68]. *DHODH* is also suppressed by miR-502, which is expressed at significantly lower levels in human colon tumors relative to normal tissue [71]. Finally, suppression of DHODH also impairs the function of complex III in the ETC, causing accumulation of p53 and induction of apoptosis, which further relates mitochondrial respiration to cancer growth and survival [72].

Purine nucleotide synthesis requires nitrogen from aspartate and glutamate as well as glycine and formate for backbone synthesis (Figure 3). While enzymes involved in glycine and formate synthesis are present in both the cytosol and mitochondria, increasing evidence suggests that the formate (and potentially glycine) fueling this pathway is primarily derived from mitochondrial metabolism. Formate is incorporated into purines via 10-formyl-tetrahydrofolate (10-CHO-THF) and thymidine via 5,10-methylene-THF. These substrates can be generated in both the cytosol and mitochondria via serine hydroxymethyltransferase (SHMT), methylenetetrahydrofolate dehydrogenase (MTHFD), and downstream reactions in folate-mediated one carbon metabolism (FOCM) [73]. We recently developed a system for quantifying the contribution of different substrates to the mitochondrial and cytosolic NADPH pools using [^2^H] tracing and inducible expression of mutants IDH1 and IDH2 [74]. Application of [^2^H]-labeled serine, glycine, and glucose tracers to non-small cell lung cancer cells indicated that serine flux through SHMT2 and MTHFD2(L) operates primarily in the oxidative direction to produce mitochondrial NAD(P)H in these cancer cells [74]. Additional evidence by others supports the concept that mitochondrial FOCM is an important contributor of reducing equivalents and one carbon intermediates for nucleotide biosynthesis [75,76]. While the cytosolic pathway may independently contribute to nucleotide biosynthesis [77], our results correlate with the recent demonstration that MTHFD2 expression is commonly elevated in many cancers and associated with poor survival in breast cancer patients [54].

## Conclusions

Mitochondria operate as both engine and factory in eukaryotes, coordinating cellular energy production and the availability of fundamental building blocks that are required for cell proliferation. Cancer cells must therefore balance their relative bioenergetic and biosynthetic needs to grow, proliferate, and survive within the physical constraints of energy and mass conservation. In contrast to quiescent cells, which predominantly use oxidative mitochondrial metabolism to produce ATP and uptake glucose at much lower rates than proliferating cells, tumor cells exhibit increased glycolytic rates to provide an elevated flux of substrate for biosynthetic pathways, including those executed within mitochondria. Given these higher rates of nutrient utilization, metabolic flux through mitochondrial pathways and the associated ROS production can often be higher in cancer cells. Not surprisingly, activation of cellular antioxidant response pathways is commonly observed in cancer or subpopulations of cells within tumors [46,78]. Cellular compartmentalization affords a degree of protection from such damaging side products of metabolism, and methods which are able to deconvolute the relative contributions of each cellular compartment (e.g. mitochondria, cytosol, peroxisome, etc.) to cancer metabolism will be crucial to more completely understand the metabolism of cancer cells in the future [74,79]. Ultimately, while mitochondrial dysregulation is widely considered to be a hallmark of cancer, numerous mitochondrial functions remain critical for tumor growth and are emerging as clinical targets.

Following this point, it comes as no surprise that mitochondrial metabolism is highly active in virtually all tumors (i.e., cancer cells, stroma, or both), and investigators have begun targeting these pathways to explore potential efficacy. Indeed, some evidence suggests that biguanides such as metformin or phenformin may limit tumor incidence and burden in humans and animals [80,81]. These effects are presumably due, at least in part, to complex I inhibition of the ETC, which significantly perturbs mitochondrial function [82,83]. However, more insights are needed into the mechanisms of these compounds in patients to determine the therapeutic potential of targeting this and other components of mitochondria. In developing new therapies that target cancer metabolism, researchers will face challenges similar to those that are relevant for many established chemotherapies since deleterious effects on normal proliferating cells that also depend on mitochondrial metabolism (and aerobic glycolysis) are likely to arise.

As we acquire a more detailed picture of how specific genetic modifications in a patient’s tumor correlate with its metabolic profile, opportunities for designing targeted or combinatorial therapies will become increasingly apparent. Cancer therapies that address tumor-specific mitochondrial dysregulation and dysfunction may be particularly effective. For example, some cancer cells harbor mutations in TCA enzymes (e.g., FH, SDH, IDH2) or regulatory proteins that control mitophagy (i.e., LKB1) [84]. Such tumors may be compromised with respect to some aspects of mitochondrial biosynthesis and dependent on alternate pathways for growth and/or survival such that synthetically lethal targets emerge. Ultimately, such strategies will require clinicians and researchers to coordinate metabolic, biochemical, and genetic information in the design of therapeutic strategies.

## Abbreviations

FDG: 2-deoxy-2-[^18^ F]fluoro-D-glucose
CH_2_-THF: 5,10-methylene tetrahydrofolate
10-CHO-THF: 10-formyl-tetrahydrofolate
ATP: adenosine triphosphate
GOT: aspartate transaminases
ASNS: asparagine synthetase
AML: acute myeloblastic leukemia
AcCoA: acetyl-coenzyme A
Ala: alanine
GPT: alanine transaminases
aKG: alpha-ketoglutarate
BCAT: branched chain aminotransferase
Cit: citrate
cALL: childhood acute lymphoblastic leukemia
P5CS: delta-1-pyrroline-5-carboxylate synthase
DHODH: dihydroorotate dehydrogenase
ETC: electron transport chain
EGF: epidermal growth factor
FOCM: folate-mediated one carbon metabolism
Fum: fumarate
FH: fumarate hydratase
Gln: glutamine
Glu: glutamate
Gly: glycine
GS: glutamine synthetase
GLS: glutaminase
GDH: glutamate dehydrogenase
HOTs: human orthotopic tumors
HIF: hypoxia inducible factor
ICT: isocitrate
IDH: isocitrate dehydrogenase
Lac: lactate
LKB1: liver kinase B1
MS: mass spectrometry
mTORC1: mammalian target of rapamycin complex 1
ME: malic enzymes
Mal: malate
MTHFD: methylenetetrahydrofolate dehydrogenase
NADH: nicotinamide adenine dinucleotide, reduced
NADPH: nicotinamide adenine dinucleotide phosphate, reduced
NNT: nicotinamide nucleotide transhydrogenase
NMR: nuclear magnetic resonance
OAC: oxaloacetate
PET/CT: positron emission tomography integrated with computed tomography
PDAC: pancreatic adenocarcinoma
Pyr: pyruvate
PDH: pyruvate dehydrogenase
PC: pyruvate carboxylase
PDK: PDH kinase
P5C: pyrroline-5-carboxylate
PYCR: pyrroline-5-carboxylate reductase
POX: proline oxidase
ROS: reactive oxygen species
Ser: serine
SHMT: serine hydroxymethyltransferase
SREBP1: sterol regulatory element binding protein 1
Suc: succinate
SDH: succinate dehydrogenase
TCA: tricarboxylic acid
TRAIL: tumor necrosis factor-related apoptosis-inducing ligand
